# Diagnosis of Herpes Simplex Virus: Laboratory and Point-of-Care Techniques

**DOI:** 10.3390/idr13020049

**Published:** 2021-06-02

**Authors:** Peuli Nath, Md Alamgir Kabir, Somaiyeh Khoubafarin Doust, Aniruddha Ray

**Affiliations:** Department of Physics and Astronomy, University of Toledo, Toledo, OH 43606, USA; Peuli.Nath@utoledo.edu (P.N.); MdAlamgir.Kabir@rockets.utoledo.edu (M.A.K.); Somaiyeh.KhoubafarinDoust@rockets.utoledo.edu (S.K.D.)

**Keywords:** herpes simplex virus, detection, diagnostics, point-of-care devices, microfluidics, imaging and microscopy

## Abstract

Herpes is a widespread viral infection caused by the herpes simplex virus (HSV) that has no permanent cure to date. There are two subtypes, HSV-1 and HSV-2, that are known to cause a variety of symptoms, ranging from acute to chronic. HSV is highly contagious and can be transmitted via any type of physical contact. Additionally, viral shedding can also happen from asymptomatic infections. Thus, early and accurate detection of HSV is needed to prevent the transmission of this infection. Herpes can be diagnosed in two ways, by either detecting the presence of the virus in lesions or the antibodies in the blood. Different detection techniques are available based on both laboratory and point of care (POC) devices. Laboratory techniques include different biochemical assays, microscopy, and nucleic acid amplification. In contrast, POC techniques include microfluidics-based tests that enable on-spot testing. Here, we aim to review the different diagnostic techniques, both laboratory-based and POC, their limits of detection, sensitivity, and specificity, as well as their advantages and disadvantages.

## 1. Introduction

Herpes simplex viruses 1 and 2 (HSV-1 and HSV-2) are DNA-based viruses from the *Herpesviridae* family, responsible for causing herpes (genital or oral) and fulminate encephalitis in humans [1,2,3,4,5]. It has been estimated that more than 500 million people globally, including 50 million in the US, have been infected with HSV [6,7,8,9]. This staggering number of positive cases worldwide can be attributed to the highly infectious nature of this virus. The virus is transmitted to a seronegative individual via abraded skin or mucosal surface. Upon entering the host body, the virus can stay dormant, generally located in the axons of the peripheral nervous system neuron. Upon experiencing proper stimulus, it travels through the epithelial cells, causing characteristic symptoms like genital lesions, oral ulcers, and blisters [4,5,6,10,11]. Studies have shown that HSV can evade the immune system and mediate cell-to-cell propagation [12,13,14]. Immune deficient HIV patients are at higher risk, with the chance of developing drug-resistant HSV infection [15,16]. Neonatal herpes in infants can occur upon exposure to HSV infection, mostly acquired from infected mothers, causing lifetime neurological defects and death [17,18]. Individuals who contract HSV may or may not show symptoms that increase the risk of spreading the disease unknowingly. The medical costs related to HSV have exceeded USD 500 million in the US alone in the past decade [19].

To date, there is no cure for HSV infection, except for some antiviral drugs that can reduce the severity of the symptoms [4,5,20]. Attempts at developing vaccines against HSV have not yet been successful due to the complex host–pathogen interaction. Early diagnosis of the infection can help patients with proper disease management and also lower the risk of transmission. Disease management studies have shown the importance of early testing and prior knowledge of patient history in guiding the proper course of treatment as well as providing counseling to patients and their significant others [2,21].

In this review, we focus on the different diagnostic technologies for herpes. These include conventional methods as well as the different advanced technologies, particularly the POC [7,22,23,24]. Diagnosis of HSV typically involves detecting the whole virus or viral proteins, genetic materials, or HSV-specific antibodies in the blood. The conventional diagnostic strategies include viral culture, serological tests, and molecular techniques [25,26]. Viral culture involves extracting the virus from the specimen, such as swab or needle aspiration, and culturing them for a few days, followed by microscopic analysis for determining HSV cytopathic effects (CPE) [26]. This type of test required high-quality specimen collection, with proper handling and transportation of the specimen. Molecular diagnosis of the virus can be achieved by nucleic acid amplification using polymerase chain reaction (PCR) [27] and loop-mediated isothermal amplification (LAMP) [28]. This method is extremely sensitive and specific in detecting the virus. Immunological assays include enzyme-linked immunosorbent assays (ELISA) [29] and Western blot assays [30] for effective detection of antigens (HSV glycoproteins) or antibodies specific to HSV glycoproteins, which have proven to be an excellent marker for HSV infection. Other methods include immunofluorescence assays [31], luminescence assays [32], and advanced microscopic techniques [26,33,34,35,36]. All these methods have their own advantages and drawbacks [37].

We also focus on the different POC devices for herpes diagnosis that are portable, cost-effective, and can be easily operated without the need for expert handling [7,23]. These devices can be used for testing the patient at home, with no or minimal supervision, which is particularly useful in remote locations and resource-limited settings. The POC tests are usually based on microfluidic platforms [24] and employ tests based on ELISA, immunofluorescence, PCR, and LAMP. Several such kits are commercially available [38,39,40,41]. The POC devices not only enable rapid and early HSV detection but are also capable of specifically differentiating the type of virus (HSV-1 vs. HSV-2).

## 2. Detection of HSV in Lesions

Traditional diagnosis of HSV from lesions involves the direct detection of tissues or cells infected by the virus, viral proteins, whole virus, or genetic materials. The virus is collected from lesions on skin or genitals by swabbing or scraping with a scalpel. This is followed by detection using the various techniques discussed in the following sections.

### 2.1. Microscopy and Imaging

**Figure 1:**
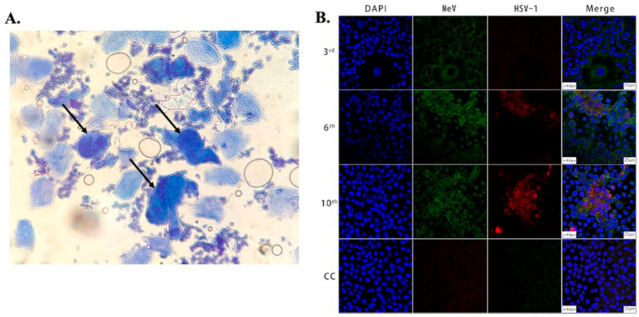
(**A**) Microscopic image showing large multinucleated giant cells (indicated by arrows) surrounded by multiple normal cells in an HSV-infected patient. The cells were stained with methylene blue, taken using an optical brightfield microscope [45]. (**B**) Images showing Vero/hSLAM cells coinfected by MeV and HSV-1 from different passages and a cell control (3rd, 6th, 10th, CC). As indicated in the 2nd and 3rd columns, the cells were subjected to immunostaining using antibodies against MeV M (green) and HSV-1 ICP0 (red) protein, and images were captured using laser scanning confocal microscopy at 40× magnification using a Leica TCS SP8 confocal microscope (Leica Microsystems; Wetzlar, Germany). The cell nuclei were stained with DAPI [68].

Brightfield Microscopy: Historically, the most widely used HSV diagnostic tests were based on Brightfield microscopy [25,26]. Brightfield microscopy involves imaging the transmitted light after passing through the specimen or the back-reflected light. The morphological information is obtained due to the attenuation of light because of absorption and/or scattering from the sample. The scattering and absorption from viral particles, which are <150 nm in size, are minuscule and, thus, individual viral particles cannot be directly observed using this modality [42]. However, brightfield microscopy can be used to image cells and tissues infected with the virus. The specimen for this method is collected by scraping the lesion (Tzanck smears) using a scalpel blade and transferring the sample to a glass slide, followed by staining with Giemsa, methylene blue, or toluidine blue [43,44,45]. These stained tissues are imaged in order to detect HSV cytopathic effects. Figure 1A shows a Tzanck smear stained with methylene blue of a sample collected from the vesicles of an HSV-infected patient, showing multinucleated giant cells under brightfield microscopy. This method is simple, inexpensive, and can be used for a wide variety of clinical specimens. However, the major drawback of this method is its sensitivity (84%), which depends on proper sample preparation, which is complex. Additionally, a large amount of tissue is needed for accurate identification, and this can be painful for patients. Secondly, the accuracy of this method depends on the stage of lesions [46,47,48]. Another drawback is its lack of specificity in differentiating HSV-1, HSV-2, and varicella-zoster virus (VZV).

In addition to tissues, the presence of the virus in swabs can be detected by culturing them in susceptible cell lines such as Hep-2, A549, MRC-5, WI38, Vero, rhabdomyosarcoma cells, rabbit kidney, and human diploid fibroblasts [26]. The changes in cell morphology, such as cytoplasmic granulation, cell rounding, or lysis, indicate the presence of the virus. The process of specimen collection and transport is very sensitive as viral envelopes are extremely labile. Contamination of the samples with bacteria or fungus can be an issue and, thus, antibiotics are used in the transport media. The sensitivity of HSV detection depends on the cell line, e.g., rabbit kidney shows 100% sensitivity even at low viral inoculum [49,50,51]. This process typically requires 5–14 days to generate the result and lacks specificity in differentiating HSV-1 and HSV-2; it also requires extreme care in collecting, handling, and transporting the specimen, which may have an effect on the test result. 

Fluorescence Microscopy: In fluorescence microscopy, the fluorescently labeled specimen is illuminated with light of a specific wavelength, which is absorbed by the fluorophore, and results in the emission of another photon with lower energy (longer wavelength), which is captured by the photomultiplier tubes (PMT) or cameras in the microscope [52]. In order to visualize the virus using fluorescence microscopy, it needs to be stained with fluorescent molecules [53,54,55]. The most widely used method of visualizing the virus using fluorescence microscopy is direct immunofluorescence assay (DFA). DFA uses specific targeting moieties, such as antibodies, tagged with fluorophores to stain the virus [52,56,57,58,59,60,61]. These antibodies typically target the glycoproteins present on the surface of the viral particles. A comparison between the efficiency of fluorescence microscopy and viral culture for detection of HSV was performed by Caviness et al. in pediatric patients [62]. In this study, samples were collected by swabbing and scraping the base of a skin lesion of the infected patients. The sample obtained was then rubbed onto glass microscope slides, air-dried, fixed, and made to react with the commercially available monoclonal antibodies labeled with fluorescein isothiocyanate (a fluorescent probe specific to HSV type 1 and 2) to form an immunocomplex. The emission of green fluorescence from the cells indicated the presence of HSV infection, as observed under a fluorescence microscope. The turnaround time of this assay was about 60–90 min. The sensitivity of the DFA method in clinical samples was found to be 61% compared to viral culture, using HFF and A549 cell lines, and the specificity was greater than 90%. However, there can be an issue of cross-reactivity with the use of monoclonal antibodies, and this might give rise to false- negative or false-positive results. Another disadvantage is the requirement of clean microscopic glass slides with a controlled thickness that may not be available in resource-limited settings.

Confocal microscopy: Fluorescence confocal microscopy, which has a higher spatial resolution compared to conventional fluorescence microscopes, has been used to study viral cytopathology [52,63,64,65,66,67]. Confocal microscopes use a spatial slit/pinhole to eliminate the out-of-focus fluorescence photons during image formation, thereby increasing spatial resolution. Li et al. recently reported the detection and characterization of HSV-1 infection from throat swabs in measles patients [68]. Serological assay (ELISA) and RT-PCR were performed to confirm and characterize the coinfection, evaluating the presence of IgG and IgM against HSV and measles virus and viral nucleic acid, respectively, in patient samples. Confocal laser scanning microscopy was used to demonstrate the dynamic of measles and HSV-1 coinfection in the Vero/hSLAM cell line using an indirect immunofluorescence assay (IFA) to stain the virus with fluorescent-labeled antibodies. The throat swab samples from confirmed measles patients were used to infect Vero/hSLAM cells for propagation. After a few days of incubation, the infected cells were extracted at different passage times, washed, and fixed on slides. After fixing, diluted antibodies specific to measles (rabbit Mev matrix protein) and HSV-1 (mouse anti-HSV-1 ICP0) were added, which formed the immunocomplex. A fluorescent-labeled secondary antibody (GFP against Mev matrix protein and RFP against HSV-1 ICP0 protein) was added to the sample to fluorescently stain the virus. The nucleus of the cells was stained with DAPI and imaged using LASER scanning confocal microscopy, as shown in Figure 1B. Mev infection was detected using the antibody for the anti-Mev matrix protein, indicated by green fluorescence, whereas HSV-1 was detected using an anti-HSV ICP0 antibody, indicated by red fluorescence. It was observed that fluorescence (red) due to HSV-1 appears strong only after the 6th passage and gradually increases thereafter, whereas the green fluorescence (Mev) decreases. This study, conducted on 40 measles patients, suggests Mev as a primary infection, whereas HSV-1 infection was due to the reactivation of the latent virus in most cases. In another study, Cinotti et al. reported the identification of HSV infection by skin-dedicated ex-vivo fluorescence confocal microscopy using fluorescent antibodies [66]. In this study, HSV samples were collected from skin lesions of infected patients and incubated with anti-HSV1 and anti-HSV2 antibodies tagged with fluorescein isothiocyanate for the identification of the virus.

Reflectance confocal microscopy (RCM) is another promising noninvasive skin imaging technique [69,70] used to study the histopathology of cells for diagnosing various skin diseases. It enables the imaging of the epidermis and papillary dermis directly, with high resolution at the cellular level without altering the skin surface. RCM imaging of a patient experiencing pustular eruptions showed the characteristics of large lobated multinucleated cells and acantholysis that are similar to the herpes-infected cytopathic effect on skin cells, and the findings were validated by the Tzanck cytodiagnosis method. These microscopic techniques are sensitive to light and motion and have a small field of view, which severely limits the throughput of the tests. A large number of images need to be acquired to obtain statistically relevant data so as to achieve results with a high degree of accuracy. In addition, these microscopes are expensive and involve complex instrumentations, making the instrument bulky and not readily available in resource-limited settings.

Transmission Electron Microscopy (TEM): The electron microscope (EM) uses a thin monochromatic beam of electrons that is focused on the sample using a magnetic lens operating at a high voltage of 200 kV. The transmitted electron beams are used to form an image and extract the structure and morphology of the samples. Unlike optical microscopy, electron microscopes can image the samples at extremely high magnifications of up to 1,000,000×, which enables high-resolution imaging of individual HSV viral particles [34,35,71,72,73]. Folkers et al. demonstrated the use of electron microscopy to diagnose HSV infection and compared its efficiency with viral culture and the Tzanck smear method [71]. For this study, the sample was collected from the base and edges of the lesion and adsorbed on a carbon-coated TEM grid. The sample was fixed with 1% glutaraldehyde, washed, and stained with 2% phosphotungstic acid (PTA) in order to observe the viruses at 10,000× magnification. Another technique is solid-phase immunoelectron microscopy, which involves viral capture using a bilayer of protein A and an antibody. The grid is first treated with protein A, followed by the immobilization of rabbit IgG anti-HSV. The grid is then washed and incubated with a drop of viral suspension, further washed with saline buffer, fixed with 1% glutaraldehyde, and stained with 2% PTA, which enables direct imaging of the virus.

Indirect labeling is another technique that involves capturing the virus using a monoclonal antibody specific to HSV-1 and HSV-2, followed by labeling with gold-tagged rabbit anti-mouse antibodies. The virus samples are prepared by collecting the lesions and homogenizing them with ultra-fine sterile quartz sand particles. This homogenized sample is centrifuged, and the virus particles present in the supernatant are used for testing [71]. The sensitivity of the EM technique has been found to be 96% compared to viral culture and the Tzanck smear method. ImmunoEM has better specificity in differentiating HSV-1, HSV-2, and VZV from skin lesions. The requirement of bulky and expensive instruments and its low throughput, which are the major drawback of this technique, have limited its utilization. Nowadays, these techniques are largely replaced by immunofluorescence based assays that can provide more sensitive type-specific detection of HSV.

Digital Holographic Microscopy: Digital holographic microscopy (DHM) is an interferometric technique that has been extensively used for the detection and imaging of biological samples [36,74,75,76,77,78]. In this type of computational microscopy, the images are digitally reconstructed from holograms, which result from the interference between the scattered optical wave and the non-diffractive wave [79]. Typically, a partially coherent light source is used to illuminate the sample, and the transmitted light (holograms) is recorded using a CMOS imaging chip placed close (<1 mm) to the sample. The holograms are then backpropagated to the object plane in order to reconstruct the images. The images contain both the phase and amplitude information. Several different algorithms can be used to reconstruct the images. For example, the widely used angular spectrum approach involves multiplying a transfer function with the Fourier transform of the captured hologram and then taking the inverse Fourier transform of the product to retrieve the image [80]. The absence of lenses enables imaging over a wide field of view, giving this technique higher throughput compared to conventional lens based microscopy techniques. Additional advantages include low cost, portability, and ease of use, thereby making this technique a promising candidate for POC detection of HSV.

**Figure 2:**
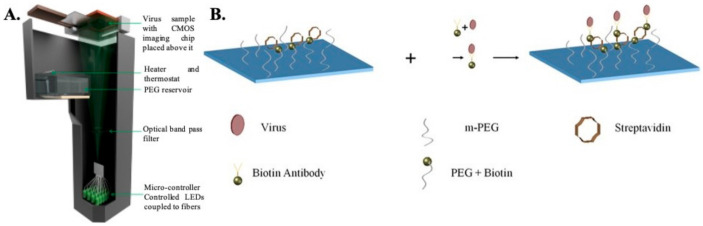
(**A**) Schematic representation of lensless holographic microscope. (**B**) Step-wise process showing attachment of the virus to the surface substrate. Firstly, the chip for specific capture of HSV-1 particles is prepared by coating a glass substrate with streptavidin and poly-ethylene-glycol. In the second step, HSV-1 particles in solution are conjugated with biotin-tagged antibodies and added onto the substrate; in the third step, HSV-1 particles captured on the substrate are then imaged by the computational holographic microscope for counting their density (counts/mm^2^) [36].

Detection of the whole virus using DHM is performed by first capturing the virus on a specially prepared substrate using monoclonal antibodies (as shown in Figure 2). The virus samples are first mixed with biotinylated antibodies to form an antibody–antigen complex and then incubated on a glass substrate coated with streptavidin. Once the virus is immobilized onto the glass surface due to the biotin–avidin bond, the samples are thoroughly washed and dried prior to imaging. Nonspecific binding is minimized by coating the bare glass surface with poly-ethylene glycol. The viruses are directly detected using DHM by creating localized liquid droplets, called nanolenses, specifically around the virus. These nanolenses help to amplify the signature of the virus, which would otherwise be too weak to detect. The nanolenses are created by depositing PEG vapor on the substrate containing the virus. The temporal evolution of the nanolenses is also used to estimate the size of the virus (~150 nm), which is then used to distinguish the viral particles from nonviral “junk” particles. The limit of detection (sensitivity) of this method has been calculated to be ~4 copies per mm^2^ of the substrate imaged or ~160 copies/test [36]. Another approach that utilizes a new method of creating nanolenses using acoustic actuation was used to detect the whole viral particles in solution, thereby expanding the capabilities of DHM for detecting HSV in both dry state and in solution [81].

A separate microparticle aggregation assay was also used to detect HSV using DHM. In this approach, HSV-specific-antibody-coated polystyrene microparticles were added to the virus sample and imaged using DHM [82,83]. The presence of the virus and the viral antigen led to clustering of the microparticles, which were quantified using a deep-learning-based algorithm. The degree of clustering was used as a metric to detect the presence of the virus and its concentration. The sensitivity, i.e., the limit of detection for this approach, was ~5 viral copies/μL (i.e., ~25 copies/test). The specificity of these techniques depends on the type of antibodies used, their antigen-binding affinity, and their rates of association and dissociation.

### 2.2. Detecting Viral Glycoproteins

Agglutination Assay: Agglutination assays are simple assays that involve the clustering of microparticles due to immunogenic reactions in the presence of the target antigen or antibody. These assays are binary, and the outcome of this test can most often be observed by the naked eye. One of the most widely used assays is based on latex microparticles [84,85,86,87]. For latex agglutination assays, swab samples are collected from infected patients and the test sample is prepared using specimen buffers, e.g., the commercially available latex agglutination test (Virogen Herpes Slide Test, Wampole Laboratory, Cranbury, NJ). After gently mixing the solution by stirring and rotating, the latex particles coated with HSV-specific antibodies are added to the test well, with the control latex (anti-human IgG) in another well. The positive samples usually form small clumps or agglutination with a cloudy background, which can be easily detected with the naked eye. The assay showed 100% sensitivity and 89% specificity, as confirmed by the viral culture of the same sample.

Western Blot Assay: In Western blot assays, the viral proteins are separated and identified using gel electrophoresis. The viral proteins are first extracted from the cells or tissue lysates collected from the patients and loaded onto a gel for electrophoretic separation with a load marker such as bromothymol blue or Coomassie blue. The separated band of proteins in the gel are then transferred onto a membrane (PVDF or nitrocellulose), called the “blotting process”. The presence of a particular target protein is detected with the addition of a fluorescent or radioactive isotope-labeled antibody specific to the protein. The location of the target protein is found out using a standardized protein ladder with a specific molecular weight [88]. The Western blot assay is considered the “gold standard” for the detection of type-specific HSV antibodies and the differentiation of HSV infections, which is discussed in Section 3.2.

### 2.3. Detecting Viral Genetic Material

**Figure 3:**
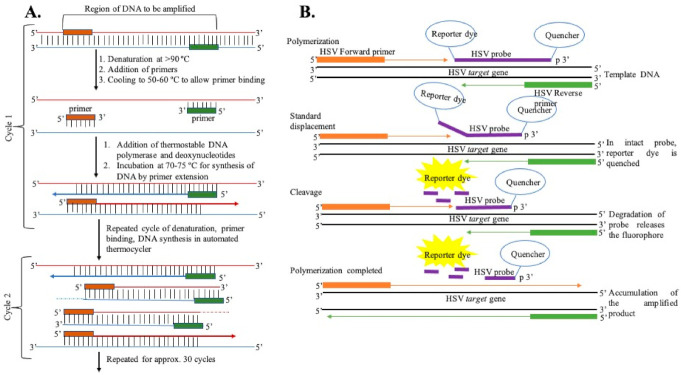
(**A**) General representation of steps of DNA amplification using polymerase chain reaction. (**B**) DNA amplification using the TaqMan chemistry mechanism.

Polymerase Chain Reaction: The most common and standard molecular diagnosis technique is polymerase chain reaction (PCR), which allows the rapid amplification of viral genomes and can be used in clinical laboratories for the detection of HSV, as shown in Figure 3 [27,89,90,91,92,93,94,95,96,97]. The PCR method, involving the use of either a TaqMan probe or the HydProbe system that targets specific sequences of the genome, undergoes several thermal cycling processes of amplification and generates the result in the form of fluorescence signal readouts; gel electrophoresis can also be used. The advantages of the fluorescence-based real-time PCR technique have completely revolutionized the PCR-based system for the quantification of DNA [98,99]. This PCR technique is highly sensitive, has high precision, eliminates any post-amplification handling, and can be operated automatically. A fluorescence dye (SYBR green) or TaqMan probe is used in real-time PCR to monitor DNA amplification as the reaction progresses. Over the years, different PCR assays have been used for the detection of HSV [97,100,101,102,103,104,105]. Marshall et al. have detected HSV from genital infections using a multiplex PCR assay [106]. For this study, patient samples were collected, and DNA was extracted for the assay. Primers specific to the TK3 gene for HSV-1 and the POL gene for HSV-2 were chosen and incubated with the genomic DNA using the Taq polymerase method. After several thermal cycling steps, the amplified DNA was subjected to the gel-electrophoresis technique for HSV detection and typing. They were successful in detecting HSV infection in four suspected patients who were shown as negative with the viral culture method. This result indicates the sensitivity of PCR in HSV typing for diagnosing genital herpes.

Recently, multiplexed quantitative PCR (qPCR) was used to detect HSV in intraoral commensal by Yap et al. [107]. For this study, the DNA was extracted from samples collected from oral swabs and saliva samples and prepared using commercially available sample processing kits. This extracted fragment of DNA was amplified using qPCR with a fluorescent probe, which is useful for monitoring the real-time amplification of HSV-1 and HSV-2 DNAs. UL44 (HSV-1) and UL3 (HSV-2) target regions were chosen for the amplification. The limit of detection for each target was established using Vircell Quantified Amplirun DNA controls and was found to be 15 cp/PCR for HSV-1 and 8 cp/PCR for HSV-2. The viral load quantification was done with a standard curve. Among a pool of samples tested for the human herpes virus, 4.3% were reported to have HSV-1 with no trace of HSV-2 DNA.

A comparative study between PCR and viral culture methods showed 100% sensitivity and specificity for the PCR technique in detecting HSV-1 and -2 over the viral culture method, which has a sensitivity of 50% with 100% specificity [108]. Another study compared the shell vial culture method and custom-designed FRET (fluorescence resonance energy transfer) based real-time- PCR, called LightCycler PCR, and found 100% specificity of LightCycler PCR, with increased sensitivity over shell vial techniques in detecting the virus [109].

Loop-Mediated Isothermal Amplification Technique: DNA amplification, with high efficiency and specificity, can also be achieved using the loop-mediated isothermal amplification (LAMP) method [28,110,111,112,113,114,115,116,117,118]. It involves the use of *Bst* DNA polymerase and a set of six primers that recognize specific sequences on the target DNA. An inner primer, along with a single strand displacing DNA polymerase, initiates DNA synthesis, which is primed by an outer primer releasing a single-stranded DNA that serves as the template for DNA synthesis. The single-stranded DNA is further primed by a second inner and outer primer hybridized to the other end of the target DNA, forming a loop DNA structure; it facilitates the amplification through the extension of the loop and the annealing of primers. The amplification cycling reaction continues, with the accumulation of 10^9^ copies of the target in less than an hour. This method amplifies DNA at an optimum temperature (65 °C) without the need for several thermocycling steps; this speeds up the process, along with the use of six specific probes that increase efficiency and can be operated with cost-effective instruments in hospitals and clinics. A simple, sensitive LAMP assay was developed by Kaneko et al. for detecting HSV-1, HSV-2, and varicella-zoster virus (VZV) [28]. These viral infections show similar symptoms but require different clinical prognoses, which makes it important to differentiate these viruses. Kaneko et al. used sets of clinically isolated strains of HSV-1, HSV-2, and VZV and used the viral DNA for their experiments. Six primers were used in the LAMP assay, including two outer primers (F3, B3), a forward inner primer (FIP), a backward inner primer (BIP), and two additional loop primers (LPF and LPB) for increased amplification efficiency. Virus-specific primers were designed to target specific gene origins, UL1 to UL2 for HSV-1, US4 for HSV-2, and ORF62 for VZV. The LAMP assay was performed using a LOOPamp DNA amplification kit. A reaction mixture containing each inner primer, outer primer, and additional loop primer, *Bst* DNA polymerase, and DNA sample was incubated at 65 °C for 45 min and then above 80 °C to terminate the reaction. Each amplified product was further analyzed using the agarose gel electrophoresis technique. The specificity and sensitivity of the LAMP-amplified products were validated by digestion with a specific restriction enzyme, PstI for HSV-1 amplicons, HaeIII for HSV-2 amplicons, and AluI for VZV amplicons, and PCR using the primer sets. The limit of detection of HSV-1 and HSV-2 using the LAMP assay was as low as 10 copies/tube for all three different strains. Compared to PCR, this assay showed better specificity in differentiating HSV and VZV and, more importantly, between HSV-1 and HSV-2 with the specific primers used for the study. LAMP is more convenient due to its temperature-independent mechanism and less hardware requirement, which makes it suitable for point-of-care testing applications. However, optimization of the amplification of specific primer sets depends on the choice of polymer, design of primers, time, and ion concentrations, which makes the test labor-intensive and often leads to nonspecific amplification; this can compromise the assay’s specificity [119].

Helicase-Dependent Amplification Technique: The helicase-dependent amplification technology is similar to PCR, but in this technique, a thermostable helicase enzyme is used to separate the DNA strands instead of thermal denaturation. This enables labeled primers hybridized to DNA templates and initiates the elongation process in the presence of DNA polymerase [40,120,121]. The amplification can be operated at a single temperature of 64 °C, unlike the series of different temperatures in the case of PCR, which makes this technique suitable for point-of-care applications. Recently, the US food and drug administration (FDA) approved a test for HSV detection, called IsoAMP^®^ HSV (Biohelix Corp., Beverly, MA, USA), based on nucleic acid amplification technique for the detection of the HSV gB gene from oral and genital lesions of symptomatic patients [40,121,122,123]. The assay consists of helicase-dependent amplification (HDA) technology and a single-use detection device (Type II BESt^TM^ cassette) with a target-specific colorimetric probe. The sample specimen from the viral transport media is diluted and transferred to an amplification tube to which a master mix is added. The tube is then heated at 64 °C for 1 h to amplify the DNA and placed in the detection device to detect the amplicon. The target amplicon is fluorescein- and digoxigenin-labeled, which are captured and visualized as colored lines in the case of positive samples on a vertical flow strip within the disposable cassette. The turnaround time of the assay is less than 1.5 h. The sensitivity of this assay has been shown to be 97.1%, with a limit of detection of 5.5 and 34.1 copies/reaction for HSV-1 and HSV-2, respectively, and specificity of 93.4%; however, the major drawback of this assay is its inability to distinguish between HSV-1 and HSV-2. This assay has been replaced by a similar HDA assay, AmpliVue HSV 1 + 2 (Quidel, San Diego, CA, USA), which has 99.2% sensitivity and 99.7% specificity for HSV-1 and HSV-2, respectively [121,124,125].

A modified version of the IsoAmp assay was employed by Tong et al. to develop a rapid, portable molecular test IsoGlow HSV typing assay for effective detection of HSV-1 and HSV-2 from genital and oral lesions [126]. They used a portable fluorescence detection device, FireFly (BioHelix, Beverly, MA, USA), and cyclic probe technology for end-point detection of HSV products amplified by HDA. The FireFly instrument has onboard computer software for real-time and end-point data acquisition. The limit of detection for this assay was 10 copies/assay for both HSV-1 and HSV-2, and the sensitivity and specificity of the assay were found to be 100% and 98–100%, comparable to the ELVIS Shell vial assay (Diagnostic Hybrids, Athens, OH, USA) for HSV typing, with a turnaround time of 1 h. This technology has a great potential for type-specific detection of HSV in resource-limited setups, without the need for any expensive instrument, and can be easily formatted with other miniaturized detectors.

Recent studies by Jevšnik et al. have compared the effective detection of herpes simplex and varicella-zoster virus from skin lesions using a commercially available test, the RT-PCR (Argene, BioMerieux, Verniolle, France) test, performed on LC480 platform (Roche Applied Science, Mannheim, Germany) and the HDA assay (Solana HSV1 + 2/VZV assay, Quidel Corporation, San Diego, CA, USA). Both tests showed good agreement of 98.3% for HSV-1 and 99.3% for VZV [127]. The limit of detection of RT-PCR reagents was about 107 copies/mL. The sensitivity of Solana’s assays was 97.7–100% for HSV-1, 92.7–99.1% for HSV-2, and 91.8–100% for VZV. The specificity recorded was 96.3–98.4% for HSV-1, 94.5–97.3% for HSV2, and 95.8–98.3% for VZV. This study indicates the significance of isothermal amplification assays in the type-specific sensitive detection of HSV, which can be easily translated for POC applications.

## 3. Detection of Viral Antibodies in Blood (Serological Assays)

An indirect method of detecting HSV infection involves the detection of antibodies in blood that are produced in response to HSV. This involves isolating serum from whole blood, drawn from patients, and using one of the following approaches to detect the presence of antibodies specific to HSV.

### 3.1. Passive Agglutination or Hemagglutination Assay

In this assay, red blood cells are adsorbed with a soluble antigen on their surface and agglutinate in the presence of a patient serum sample with antibodies specific to the antigen [128,129]. It has been identified that glycoprotein C (gC-1) of HSV-1 is a major virus hemagglutinin, which has the ability to bind to red blood cells upon infection. Studies have shown that antibodies against this glycoprotein (gC-1 for HSV-1 and gC2 for HSV-2) appear at an early stage of the infection, which can be used as a potential candidate for early detection of the virus [129,130]. For this assay, red blood cells sensitized with gC-1 or gC-2 proteins are added to the microtiter plate. Diluted sera of patients, with antibodies specific to gC-1 and gC-2, bind and agglutinate the red blood cells within 3 h. The formation of a distinguished macroscopic pattern of agglutination indicates the presence of HSV infection. In the hemagglutination inhibition assay, the microtiter plate is loaded with the red blood cells, the antigen (glycoproteins), and the serum samples. The antibody present in the sample reacts with the antigen, thus inhibiting the agglutination of the red blood cells. The sensitivity of the hemagglutination assay and the hemagglutination inhibition assay was found to be 97%, with 84% specificity, and approx. 2 × 10^6^ HSV particle/mL is required for the assay to generate results.

### 3.2. Western Blot Assay

In this process, the whole HSV-1 and HSV-2 antigens from infected cell lines are separated by electrophoresis, then absorbed onto the nitrocellulose membrane and exposed to the patient serum [30,131,132,133,134,135]. The detection of HSV is based on the band patterns formed specifically to HSV-1 and HSV-2. The presence of specific glycoprotein G (gG) differentiates HSV-1 from HSV-2 infections, namely, gG-1 for HSV-1 and gG-2 for HSV-2. The commercially available Western blot assay Anti-HSV-1/HSV-2-gG-2 EUROLINE-WB (EUROIMMUN^®^, Germany) has nearly 98% sensitivity and specificity between 65.4% to 100%, depending on the variability of the population and the location of sample collection [131,136]. Although the Western blot technique is accurate in detecting HSV, it can generate false-negative results for HSV-2 infection if the patient has tested seropositive for HSV-1. It is also expensive and time-consuming, which are the major disadvantages of this assay.

### 3.3. Enzyme-Linked Immunosorbent Assay (ELISA)

**Figure 4:**
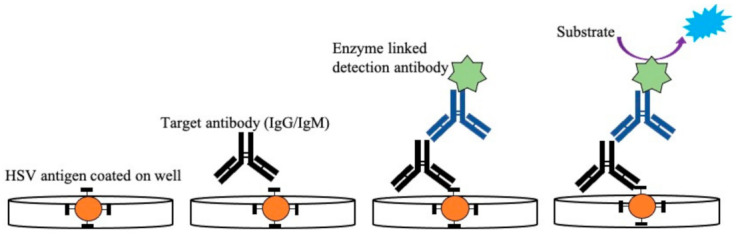
Illustration of enzyme-linked immunosorbent assay used for detection of HSV-2 IgG/IgM from patient samples. The antigen specific to IgG/IgM was coated on a microtiter plate followed by the addition of serum samples with the target antibody (IgG/IgM). After the formation of the Ag–Ab immunocomplex is detected, the anti-antibody specific to IgG/IgM linked with the enzyme is added, which subsequently binds to the target antibody. After the addition of a specific substrate, a colored compound is formed. This color change is proportional to the antibody titer present in the sample and is read by a microplate reader.

Detection of antibodies from patients’ blood using whole antigens can also be achieved using ELISA, a standard serological method [29,137,138,139,140,141]. ELISA is easier to perform and generates results faster than Western blot assays [131]. The principle behind the detection of HSV using this technique is to determine the presence of HSV-1 and HSV-2-specific IgG antibodies. The type-specific antigen, obtained from cell lysates, is coated on a microtiter plate, including a control. The diluted serum sample collected from the infected patient is then added to the well to form the immunoconjugates, as shown in Figure 4. A secondary biotinylated anti-goat IgG immunoglobulin tagged with the Avidin enzyme is added to form the antigen-antibody-antiantibody complex. Any unconjugated molecules are removed by repeated washing. Then, a specific chromogenic substrate solution is added, which forms a colored compound. Absorption of the formed colored compound is read by an automatic ELISA reader to quantify the antibody titer. Some of the commercially available ELISA tests use whole antigens, including Diamex Immunosimplicity HSV, HSV-1 or -2 IgG by Inverness, and HSV-1 or -2 IgG enzyme immunoassay by Zeus Scientific [26,132]. The sensitivity of these assays is around 92–100%, and the specificity is around 61–85% in differentiating both HSV types. Serological tests with nonspecific binding have poor specificity. Because of this lack of specificity toward whole antigen preparation, the more recent ELISA tests are based on type-specific HSV glycoprotein G (gG) [33,140,142,143,144,145]. The developed type-specific detection tests are used to detect antibodies against specific glycoproteins (gG1 and gG2) of HSV-1 and HSV-2. Commercially available gG ELISA tests include the use of purified or recombinant gG-1 or gG-2 protein for better specificity by HerpeSelect 1 or 2 (MRL/Focus Diagnostics) and Kalon HSV-2 ELISA (Kalon Biological), with sensitivity around 69–100% and 93–100% specificity [26,141,146,147]. The first US Food and Drug Administration (FDA)-approved ELISA POC kit for HSV-2 detection was SureVue-HSV-2 from Fisher (Houston, TX, USA), formerly known as the *biokit* HSV-2 rapid test from Biokit, US [41]. The test detects the gG2 gene of HSV-2 using capillary blood and serum. The specificity of the assay was found to be 93.2% to 98.7%, and sensitivity was about 99.1%. The seroreversion of gG-specific antibodies can occur, which may even go undetected at times; this limits the long-term reliability of these tests. Therefore, there is a chance of generating false-negative results in such cases, with a possible chance of reactivation of the infection. Additionally, most of these type-specific tests are unable to detect HSV-2 antibodies in HSV-1 seropositive patients, which can affect the course of the diagnosis [142].

### 3.4. Fluorescence Immunoassay

A time-resolved fluorescence immunoassay based on the indirect assay method has been used for the quantitative determination of HSV IgG in human serum samples [148]. For this study, HSV-1 and HSV-2 antigens were coated on a 96-well microtiter plate prior to adding the serum. A fluorescent (Eu^3+^)-labeled goat anti-human IgG polyclonal antibody was used as the detection antibody. The fluorescence signal was used to quantify the serum IgG level of the patient based on a pre-prepared calibration curve. The detection range of this method was found to be 5–500 AU/mL, with a limit of detection of 0.568 AU/mL. This method is more sensitive and convenient to perform compared to ELISA platforms.

### 3.5. Multiplexed Flow Immunoassay

Automated multiplexed flow immunoassays (MFIs) allow simultaneous detection of multiple analytes in a single reaction tube [26,149]. MFI technology uses microspheres of different sizes, conjugated with capture antigen (gG1 for HSV-1 or gG2 for HSV-2). The antibodies present in the serum binds to this antigen to form a complex that is detected by a fluorescently labeled reporter molecule, whose emission is measured using a flow-based fluorescence detector. There are three FDA-approved multiplex flow immunoassays) that are used to detect and differentiate HSV-1 and HSV-2: AtheNA Multi-Lyte (Zeus Scientific, Raritan, NJ, USA), BioPlex 2200 (BioRad Laboratories, Hercules, CA, USA), and Plexus HerpeSelect (Focus Diagnostic, Cypress, CA, USA) [150]. These assays are rapid (~4 h turnaround time for 180 samples), fully automated, and have high sensitivity and specificity of 99.2% and 90.2% for HSV-1, 97.4% and 85.5% for HSV-2 for AtheNA Multi-Lyte; 99.2% and 96.2% for HSV-1, 98.3% and 97.4% for HSV-2 for BioPlex 2200; and 96.5% and 98.8% for HSV-1 and 93.2% and 98.4% for HSV-2 for Plexus HerpeSelect, respectively.

### 3.6. Luciferase Immunoprecipitation Assay

An alternative assay technique to measure antibody titers in patients is the luciferase immunoprecipitation system (LIPS) [151,152,153]. In this assay, the antigen of interest (gG1 for HSV-1 and gG2 for HSV-2) is fused with the C-terminus of *Renilla* luciferase (Ruc) and the pREN2 expression vector to form a recombinant plasmid consisting of the type-specific gG protein of HSV and Ruc and transfect into Cos1 mammalian cells for multiplication. A certain amount of Ruc-tagged antigen is obtained from cell lysate after several steps of purification and is incubated with the serum sample for measurement of antibodies in HSV-infected patients. The antibodies specific to the gG1 or gG2 protein present in the serum bind to the Ruc-tagged antigen to form the Ab–Ag immunocomplex. The immunocomplex is then captured by protein A/G beads or secondary immunoglobulins immobilized on a microtiter plate. Consequently, by adding the substrate specific for Ruc, a luminescence light is generated by the Ruc-tagged antigen–Ab complex, which is then measured by a luminometer. LIPS is a sensitive, rapid assay technique with sensitivity and specificity of 92% and 96% for HSV-1 and 100% sensitivity and specificity for HSV-2 compared to Western blot assays [151]. LIPS can be adapted to different formats, including microfluidic devices [154], rapid tests, 96-well plates, single tube assays, and arrays to collect high quantitative antibody data as well as point-of-care devices [155]. The required time to perform LIPS is less than 2.5 h, which is faster than ELISA and Western blotting.

### 3.7. Microfluidic-Based Point-of-Care Devices

A simple POC device comprises of (i) a biological recognition element (enzyme, protein, antibody, and aptamer) that selectively interacts with the antigen and (ii) a transducer that monitors the interaction and provides the outcome both qualitatively and quantitatively. The World Health Organization (WHO) has set out a specific guideline for developing point-of-care devices for resource-limited applications that promise affordable, specific, sensitive, user-friendly, rapid, equipment-free analysis and delivery (ASSURED) for “on-site” analysis of samples to enhance global healthcare quality [22,156,157].

One of the important aspects of POC devices is the ability to provide rapid analysis of the sample without any sophisticated instruments and minimal or no expert supervision. Microfluidic platforms have been widely used for POC devices due to their low cost, portability, and ability to support high yield assays. Microfluidic platforms typically utilize channels of dimension less than 1000 micron, which allow rapid diffusion of reagents and samples, laminar flow, and large surface-to-volume ratios and facilitate tests with small sample volumes [158,159,160,161]. Microchannels can be made of any suitable material, such as polymers, paper, glass, and fibers. Microfluidic technology in the form of lateral flow immunoassay (LFIA), which utilizes nanoparticles such as gold nanoparticle/fluorescent nanoparticles tagged with specific antibodies, has been used for the rapid diagnosis of HSV [162,163,164,165]. The only commercially available FDA-approved microfluidic-based POC device for the detection of HSV-2 is Uni-Gold^TM^ (Trinity Biotech Plc, Bray, Ireland) [166]. This device is small and portable and utilizes purified HSV-2-specific gG2 antigens fixed to a nitrocellulose membrane for the detection of antibodies specific to HSV-2 gG2 in human blood or serum. The device consists of a test zone with the gG-2 antigen and human IgG in the control line. The whole-blood or serum sample is added to the device, which passes through a blood separation membrane and migrates across the nitrocellulose membrane towards the test line with the help of a buffer present in a separate well. Another secondary antibody–gold nanoparticle conjugate also flows towards the test/control line and forms the Ag–Ab–Ab gold nanoparticle-conjugated immunocomplex. In the case of a positive sample, a pink line is formed in the test line and in the control line of the device. The turnaround time is approximately 15 min, and the test has been approved for general practice and reference laboratory use. The sensitivity and specificity of the device are found to be 94% and 99% when compared to the Western blot technique.

Laderman et al. developed a similar lateral flow immunochromatographic assay (LFIA) using gold nanoparticles attached with anti-HSV-2 IgG using a dual-antigen direct sandwich assay [24]. The sensitivity of the developed device was found to be 100%, and its specificity was 97.3%. This device has a clear potential for developing a POC test for HSV-2 detection.

**Figure 5:**
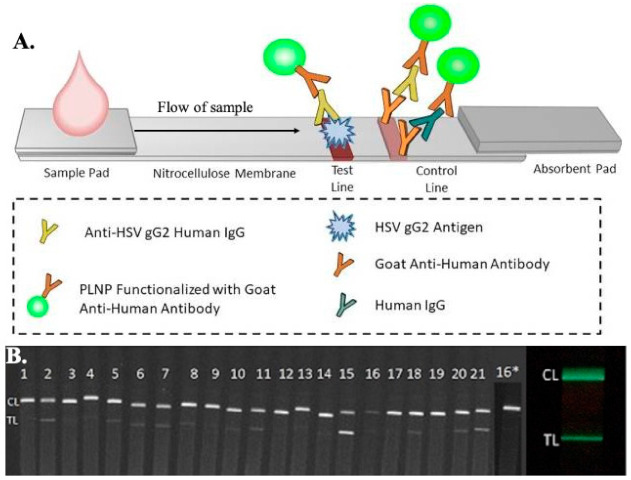
(**A**) Schematic representation of the microfluidic-based lateral flow immunoassay for the detection of antibodies present in patients using luminescent nanoparticles (PLNPs). A diluted sample was added to the sample pad, which was then mixed with PLNPs functionalized with goat anti-human IgGs to form human IgG–PLNP complexes on the LFA strip. The anti-HSV gG2 human IgG PLNP complexes migrated up the membrane and were captured by recombinant HSV gG2 immobilized at the test line. The uncaptured nonspecific human IgG-PLNP complexes were captured by goat anti-human IgGs immobilized at the control line. (**B**) Images of the serum/plasma panel tested with the HSV-2 PLNP LFA strips, captured using an iPhone 7 smartphone with a preinstalled LFA iPhone app showing phosphor emission. (Panel no 16* was re-tested at a higher dilution due to weaker control line) [167].

Another lateral flow assay test was developed by Goux et al. to detect HSV-2 infections, with enhanced sensitivity using luminescent nanoparticles (nanophosphor) tagged with goat anti-human antibodies, as shown in Figure 5 [167]. The assay strip consists of a test line of recombinant glycoprotein G from HSV-2, a control line of goat anti-human antibodies. The luminescent nanoparticles, after conjugation with the sample, are excited using a LED of a particular wavelength for 1 min. The test strip, containing the test reagents and the sample, is imaged using a FluorChem-based imaging platform and the LFA iPhone app on an iPhone 7 plus smartphone. ImageJ software is used to analyze the data. The sensitivity and specificity of the nanophosphor LFA have been found to be 96.7% and 100%, respectively, for HSV-2 detection.

In a different approach, Zubair et al. developed a miniature device for quantitative detection of antibodies for HSV-2, integrating the luciferase immunoprecipitation technique into a microfluidic platform [154]. For this assay, protein A/G is immobilized on the PDMS-glass microchannels with the simultaneous introduction of the sample and recombinant light-emitting antigens (*Renilla*, luciferase-tagged antigens) to measure the antibody titer using chemiluminescence. The total assay time for this assay is under 10 min, and it has shown 100% sensitivity and specificity for HSV-2 detection, conducted using 20 human plasma samples. Although more evaluation is still required, these devices have great potential to be fully automatic and portable test kits.

## 4. Conclusions

In this review, we have described different conventional laboratory-based detection techniques, including the agglutination assay, the viral culture method, and serological and molecular diagnosis assays used for detecting HSV infection with respect to their sensitivity and specificity in differentiating HSV 1, HSV 2, and VZV, which have different treatments compared to herpes infection. Typically, the most commonly used clinical tests from swab samples are viral culture and PCR due to their high sensitivity and specificity. In contrast, immunoassays (MFI/ELISA) are commonly used for blood tests as they can simultaneously detect multiple types of antibodies; therefore, it helps in distinguishing HSV type 1 and 2 infections, particularly in patients with unrecognized HSV infections. The accuracy of any laboratory diagnosis tests for the detection of HSV infection depends on the stage of infection when the sample is collected, the quality of the specimen, the type of tests performed, the accuracy of the method, and the interpretation of the test report by the clinician. A summary of all the techniques and their efficacies is presented in Table 1. We also focused on the different POC devices available for early herpes diagnosis, which is necessary for proper clinical prognoses and to prevent recurrence and spread of the infection. Advanced POC devices hold the key to the future of HSV diagnosis, as they would enable testing on the spot and on demand, even at home. This approach will revolutionize HSV testing and will be especially important in resource-limited settings.

## Figures and Tables

**Table 1:** Summarized list of all the detection techniques, including POC devices for the detection of HSV infection.

Techniques	Sensitivity/LOD	Specificity	Time of Assay	Reference
Detection using viral lesions				
Tzanck smear	84%	-	-	[26]
Viral culture	100%	-	5–14 days	[26]
Direct fluorescence assay	61%	90–100%	60–90 min	[62]
Immunoelectron microscopy	96%	-	-	[71]
Digital holographic microscopy (DHM)	~160 copies/test	-	3 h	[36]
Microparticle aggregation assay using DHM	~25 copies/test	-	-	[81]
Agglutination assay				
Latex agglutination assay	100%	89%	3 h	[84]
Polymerase chain reaction			>4 h	
qPCR	15 cp/PCR (HSV-1); 8cp/PCR(HSV-2)	-		[107]
RT-PCR	100%	100%		[108]
LightCycler PCR	-	100%		[109]
Loop-mediated isothermal amplification	10 copies/tube	-	75 min	[28]
Helicase-dependent amplification			1.5 h	
IsoAMP^®^ HSV (Biohelix Corp. Beverly, MA, USA)	97.10%	93.40%		[40]
AmpliVue HSV 1+2 (Quidel, San diego, CA, USA)	99.20%	99.70%		[121]
IsoGlow HSV typing assay	100%	98–100%		[126]
Solana HSV1+2/VZV, Quidel, San diego, CA, USA)	97.7–100% (HSV-1); 92.7–99.1% (HSV-2)	96.3–98.4% (HSV-1); 94.5–97.3% (HSV-2)		[127]
Detection of antibodies in blood				
Hemagglutination assay	97%	84%	3 h	[129]
Western blot assay				
Anti-HSV-1/HSV-2-gG2 Euroline-WB, Euroimmun^®^, Lubeck, Germany	98%	65.4–100%	>3 h	[131]
Enzyme-linked immunosorbent assay			2h	
using whole antigen				
Diamex Immunosimplicity HSV	100% (HSV-1 and HSV-2)	71% (HSV-1); 61% (HSV-2)		[26]
HSV-1 or-2 IgG (Inverness)	98% (HSV-1); 95% (HSV-2)	68%(HSV-1); 85% (HSV-2)		[26]
HSV-1 or-2 IgG enzyme immunoassay (Zeus Scientific, Raritan, NJ)	92% (HSV-1); 98% (HSV-2)	72% (HSV-1); 79% (HSV-2)		[26]
gG-based ELISA				
HerpeSelect 1 or 2 (Focus Diagnostics, Cypress, CA, USA)	98% (HSV-1); 100% (HSV-2)	94.1% (HSV-1); 97.1% (HSV-2)		[26]
Kalon HSV-2 ELISA (Kalon Biological, Guildford, UK)	100% (HSV-2)	100% (HSV-2)		[26]
biokitHSV-2	99.10%	93.2–98.7%		[26]
Fluorescence immunoassay	0.568 AU/mL	-	-	[148]
Multiplexed flow immunoassays			4 h for 180 samples	
AtheNA Multi-Lyte (Zeus Scientific, Raritan,NJ)	99.2%(HSV-1); 97.4% (HSV-2)	90.2% (HSV-1); 85.5%(HSV-2)		[150]
BioPlex 2200 (BioRad Laboratories, Hercules, CA)	99.2% (HSV-1); 98.3% (HSV-2)	96.2% (HSV-1); 97.4% (HSV-2)		[150]
Plexus HerpeSelect (Focus Diagnostic, Cypress, CA)	96.5% (HSV-1); 93.2% (HSV-2)	98.8% (HSV-1); 98.4% (HSV-2)		[150]
Luciferase immunoprecipitation assay	92% (HSV-1); 100% (HSV-2)	96% (HSV-2); 100% (HSV-2)	<2.5 h	[151]
Microfluidic based POC device			-	
Uni-Gold^TM^ (Trinity Biotech, Bray, Ireland)	94%	99%	15 min	[166]
LFIA	100%	97.30%		[24]
Nanophosphor-LFIA	96.70%	100%		[167]
MicroLIPS	100%	100%	<10 min	[154]

## Data Availability

Not applicable.
